# Threshold of Reactivity and Tolerance to Precautionary Allergen-Labelled Biscuits of Baked Milk- and Egg-Allergic Children

**DOI:** 10.3390/nu13124540

**Published:** 2021-12-18

**Authors:** Vincenzo Fierro, Valeria Marzano, Linda Monaci, Pamela Vernocchi, Maurizio Mennini, Rocco Valluzzi, Stefano Levi Mortera, Rosa Pilolli, Lamia Dahdah, Veronica Calandrelli, Giorgia Bracaglia, Stefania Arasi, Carla Riccardi, Alessandro Fiocchi, Lorenza Putignani

**Affiliations:** 1 Translational Research in Pediatric Specialities Area, Allergy Unit, Bambino Gesù Children’s Hospital, IRCCS, 0165 Rome, Italy; vincenzo.fierro@opbg.net (V.F.); maurizio.mennini@opbg.net (M.M.); roccoluigi.valluzzi@opbg.net (R.V.); lamiaantanios.dahdah@opbg.net (L.D.); veronica.calandrelli@opbg.net (V.C.); stefania.arasi@opbg.net (S.A.); carla.riccardi@opbg.net (C.R.); 2 Multimodal Laboratory Medicine Research Area, Unit of Human Microbiome, Bambino Gesù Children’s Hospital, IRCCS, 00165 Rome, Italy; valeria.marzano@opbg.net (V.M.); pamela.vernocchi@opbg.net (P.V.); stefano.levimortera@opbg.net (S.L.M.); 3 Institute of Sciences of Food Production, CNR-ISPA, 70126 Bari, Italy; linda.monaci@ispa.cnr.it (L.M.); rosa.pilolli@ispa.cnr.it (R.P.); 4 Department of Diagnostics and Laboratory Medicine, Unit of Allergy and Autoimmunity, Bambino Gesù Children’s Hospital, IRCCS, 00165 Rome, Italy; giorgia.bracaglia@opbg.net; 5 Department of Diagnostics and Laboratory Medicine, Unit of Microbiology and Diagnostic Immunology, Unit of Microbiomics, and Multimodal Laboratory Medicine Research Area, Unit of Human Microbiome, Bambino Gesù Children’s Hospital, IRCCS, 00165 Rome, Italy; lorenza.putignani@opbg.net

**Keywords:** labelling, food allergy, prevention, proteomics, mass spectrometry, cow’s milk allergy

## Abstract

Extremely sensitive food-allergic patients may react to very small amounts of allergenic foods. Precautionary allergen labelling (PAL) warns from possible allergenic contaminations. We evaluated by oral food challenge the reactivity to a brand of PAL-labelled milk- and egg-free biscuits of children with severe milk and egg allergy. We explored the ability of proteomic methods to identify minute amounts of milk/egg allergens in such biscuits. Traces of milk and/or egg allergens in biscuits were measured by two different liquid-chromatography-mass spectrometry methods. The binding of patient’s serum with egg/milk proteins was assessed using immunoblotting. None of the patients reacted to biscuits. Egg and milk proteins were undetectable with a limit of detection of 0.6 µg/g for milk and egg (method A), and of 0.1 and 0.3 µg /g for milk and egg, respectively (method B). The immunoblots did not show milk/egg proteins in the studied biscuits. Milk/egg content of the biscuits is far lower than 4 µg of milk or egg protein per gram of product, the minimal doses considered theoretically capable of causing reactions. With high sensitivity, proteomic assessments predict the harmlessness of very small amount of allergens in foods, and can be used to help avoiding unnecessary PAL.

## 1. Introduction

The subset of food-allergic patients sensitive to minute amounts of foods is facing problems of food safety every day [1]. To protect them from accidental ingestions, regulatory authorities have put in place legislative measures prescribing the declaration of food allergen ingredients in the respective food labels [2]. Beyond food allergen ingredients, precautionary labelling of allergens (PAL) has been adopted by food producers as additional level of protection when food allergens may contaminate foods. PAL conveys an “information on the possible and unintentional presence in food of substances or products causing allergies or intolerances, provided voluntarily by the food business operator” [3].

PAL may further reduce the possible food choices of consumers who are already forced to reduce their options [4]. Conversely, PAL-free foods may contain significant amounts of food allergens introduced by contamination at some point in the preparation chain [5]. To discipline PAL, several organisms propose the adoption of a risk-based approach [6]. According to the Voluntary Incidental Trace Allergen Labelling (VITAL) system, a voluntary scheme developed by the Australian and New Zealand food industries [7], food industry may choose to renounce to precautionary statement (action level one) when a food allergen contamination is unlikely, or to include the statement ‘…. may be present’ (action level two) depending on the circumstances. The VITAL reference doses for specific food allergens are derived from diagnostic Oral Food Challenges (OFCs) in populations of patients with food allergy. For a biscuit that may contain egg and/or milk, action level one is suggested at a reference dose of 0.2 mg total protein. For a reasonable portion size of 50 g of biscuits, this translates in a suggested exemption from PAL when the concentration is of 4 mg total protein/kg or less [8,9].

As threshold doses have been not calibrated on a population of patients with severe food allergy, but on the entire population of food allergy sufferers, the risk thresholds may be over-evaluated [10]. Although only a part of the milk/egg allergic patients are also reactive to baked foods [11], the current thresholds were derived from OFCs without distinction among baked-tolerant and baked-allergic patients. Those allergic to baked forms are considered the most reactive portion of the milk/egg allergic population [12], but their thresholds have been only rarely compared to those of milk/egg allergic patients tolerant to baked foods [13].

In this scenario, studies on the effective clinical relevance of smaller doses than the VITAL thresholds in patients extremely allergic to milk and egg are lacking. For this reason, we wanted to evaluate in a group of baked egg- and/or baked milk-allergic patients the tolerance of a baked product labelled as ‘may content little amounts of milk and egg’. A secondary objective of our study was to verify the protein quantity of milk and egg in the product using different analytical methods, in order to establish the relationship between quantities traceable below the 1% threshold and the possible development of symptoms.

Finally, this study offers us the opportunity to evaluate the threshold of reactivity to milk- and egg-baked proteins in children severely allergic to these foods.

## 2. Caseload and Methods

### 2.1. Patients

Between January 2016 and December 2019, pediatric patients aged 6 months–18 years affected by IgE-mediated milk and/or egg allergy were consecutively evaluated for their reactivity to baked milk/egg at Allergy Division of Bambino Gesù Children’s Hospital in Rome. Patients with a history of immediate (<2 h) reactions to baked milk and/or egg sensitized to baked milk and/or egg using skin prick test (SPT) and with a positive specific IgE determination for milk, egg and/or their fractions, were included. For those without a recent convincing history of anaphylaxis, a confirmatory OFC with baked milk and/or egg was required. Children with unstable asthma and severe uncontrolled eczema were included when clinically stabilized.

### 2.2. Study Design

In a monocentric, prospective design, the patients were exposed to double-blind, placebo-controlled food challenge (DBPCFC) with milk-free, egg-containing or egg-free, milk-containing biscuits for confirmation of baked egg or baked milk allergy, respectively. The usual contraindications to OFCs were applied [14]: when an anaphylactic reaction had occurred <6 months before inclusion into the study for children of 0.5 to 5 years; <12 months for children aged 6–12 years, and <2 years for adolescents, the clinical history was considered sufficient proof of allergy to baked milk or egg. Such patients were not exposed to any confirmatory DBPCFC.

Children allergic to baked proteins were tested for their tolerance to egg-free, milk-free biscuits at OFC and SPT.

### 2.3. Diagnostic Challenges

DBPCFCs performed to confirm food allergy were calibrated up to 50 g of biscuit, corresponding to 4 g of baked milk or egg proteins. We used milk-free, egg-containing biscuits (“Pavesini”, Barilla G. e R. Fratelli S.p.A., Parma, Italy; 2.09 g egg protein per 100 g product) for confirmation of baked egg allergy, and egg-free, milk-containing biscuits (“Biscottino Primi Mesi”, Plasmon, Milano, Italy; 1.30 g milk protein per 100 g product) for confirmation of baked milk allergy. The challenges were administered in seven growing doses, with an initial dose of 0.25 g, corresponding to 5.20 mg egg or 3.25 mg milk protein, respectively. We proportioned the challenge doses to the reasonable dose for each age, using a lower dose in younger children and increasing the food amount up to 45.75 g in adolescents (Table S1). The foods were blinded according to the standardized AAAAI/Europrevall protocol [15,16]. The oral challenges were discontinued at the first onset of objective symptoms [17]. Patients were observed up to 6 h after starting the test.

### 2.4. Milk/egg IgE Sensitization

SPT with cow’s milk, casein, egg white, egg yolk (Lofarma, Milano, Italy), fresh cow’s milk, fresh egg white, Pavesini, and Biscottino Primi Mesi were performed. In this case, 10 mg/mL histamine phosphate in 50% glycosaline and glycosaline on its own (Lofarma, Milano, Italy) were used as positive and negative controls, respectively. A Dome-Hollister-Stier lancet with a 1 mm tip was used for the procedure. Wheal diameters were read through a clear plastic calliper disk scaled in mm under × 4 magnification, and were interpreted when the wheal margin was included within a complete caliper circle to the nearest mm [18]. A limit of 5 mm was set for SPT positivity.

Ten mL of venous blood was collected from the patients to determine serum IgE levels (total and specific for cow’s milk proteins, casein, egg white, egg yolk) using ImmunoCAP^®^ (Thermo Fisher Scientific, Waltham, MA, USA), with 0.35 kU/L as a lower limit of eligibility [19]. Part of the serum was stored at –80 °C for successive protein sensitivity evaluations.

### 2.5. Evaluation of the Clinical Tolerance to the Biscuits Labelled without Milk and Egg

Within one month of inclusion in the study, the selected patients underwent SPT and OFC with milk and egg –free biscuits “Magretti” [Galbusera S.p.A., Cosio Valtellino (SO), Italy] from 12 different lots. Such biscuits are labelled as “not containing milk and egg”. However, the label indicates “it cannot be excluded that any traces of these allergens are present, in any case less than 5 mg/kg” (Table 1).

Based on the age of patients, seven or eight incremental doses of food were administered at 20-min intervals under clinical supervision. The first six doubling doses, starting from 0.25 g up to 8.00 g, were the same for all study participants. The seventh and eighth doses differed in different age groups (Table S1). A sample of the biscuits used for the OFC and SPT was stored for proteomic evaluation. A symptoms-based clinical score assessing the degree of gastro-intestinal, respiratory, cardiovascular and dermatological reactions was applied to monitor the acute allergic reactions. The procedure was interrupted when clear-cut objective symptoms were present, or after any severe, persistent (over 40 min) subjective symptom, according to our stopping rules [17].

### 2.6. Evaluation of the Presence of Milk/Egg Traces in Biscuits Labelled without Milk and Egg

**Figure 1:**
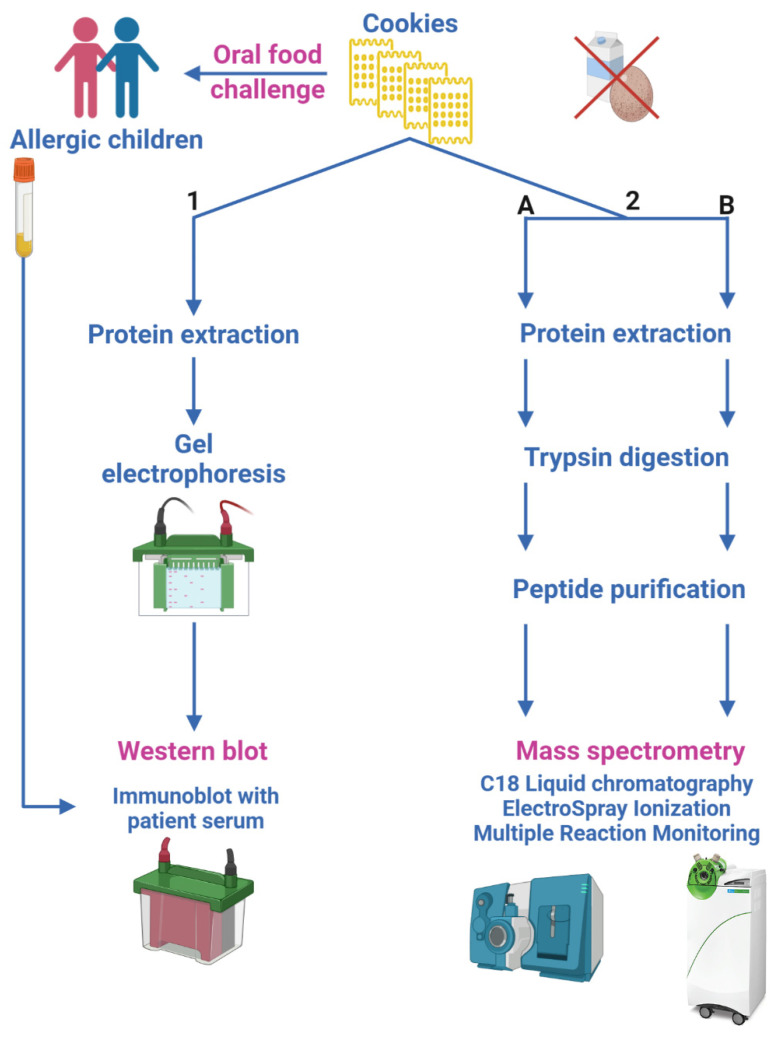
Sketch of the proteomic experimental approaches. (1) Immunoblot experiments determine patient serum binding to egg and milk allergens; (2) LC-ESI-MS/MS analyses, following two different workflows and different Triple Quadrupole MS platforms (2.A and 2.B), quantify egg and milk allergens in biscuits.

Biscuits samples used in OFC and SPT were stored at −80 °C in powder form and analyzed by immunoblot and Liquid Chromatography–ElectroSpray–tandem Mass Spectrometry (LC-ESI-MS/MS) for the detection of very small amounts of milk and egg allergens. The samples underwent the proteomic workflow for allergen detection and quantification as depicted in Figure 1. We adopted two different strategies in order to assess the contamination by egg and milk allergens:

1. immunoblot, to determine patient serum binding to egg and milk allergens possibly contained in tested biscuits (Section 2.6.1);
2. two different LC-ESI-MS/MS methods, aimed at quantifying egg and milk allergens in biscuits by monitoring their marker peptides (Section 2.6.2).

#### 2.6.1. Protein Extraction, Gel Electrophoresis Separation and Immunoblot Analysis

Biscuits were first ground coarsely using a mortar and pestle, and then milled mechanically for 30 s (three times, 10 s) in a blender (IKA Werke GmbH, Staufen im Breisgau, Germany). The biscuits’ powder was weighted and 1 g of sample was combined with 20 mL of extraction buffer (50 mM Sodium carbonate/bicarbonate pH 9.6), then it was left shaking for 2 h at 60 °C. The extract was sonicated for 5 min, 4 s pulse and 4 s pause, 60% amplitude (VibraCell Ultrasonic Liquid Processor, Sonics and Materials Inc, Newtown, CT, USA) and centrifuged for 10 min at 3000 g at room temperature (r.t.). In this case, 10 mL of supernatant were filtered through first an Acrodisc 25 mm syringe filter by a 1.2 μm Versapor membrane (Pall Corporation, Ann Arbor, MI, USA) and then by a 0.45 μm acetate cellulose membranes (Minisart Syringe Filter, Sartorius Stedim Biotech GMbh, Goettingen, Germany). Four mL of the filtrate were loaded on centrifugation filter devices [Amicon Ultra, 3000 Da molecular weight cut-off (MWCO); Merck Millipore, Billerica, MA, USA)], and concentrated 10 times. Protein concentration of the filtered and concentrate sample was determined with the colorimetric Bicinchoninic Acid Assay (Thermo Fisher Scientific). One-dimensional sodium dodecyl sulfate-polyacrylamide gel electrophoresis (SDS-PAGE) of samples was performed loading 40 μg of extracted proteins onto 12% Bis-Tris Criterion XT precast gels (11 cm, Bio-Rad Laboratories, Hercules, CA, USA); separation was performed in running buffer (25 mM Tris, 192 mM Glycine, 0.1% SDS) on a Criterion Cell apparatus (Bio-Rad) at 120 V.

Immunoblotting was performed by transferring proteins from gel to polyvinylidene fluoride membrane (Bio-Rad) at 350 mA for 2 h, in a cold room at 4 °C, on Criterion™ Blotter with wire electrodes (Bio-Rad) in presence of transfer buffer (25 mM Tris, 192 mM Glycine, 20% Methanol). The membranes were left in contact with a solution of Pierce™ Protein-Free T20 Blocking Buffer (Thermo Fisher Scientific) and then incubated overnight at 4 °C in serum of each study subject, diluted 1:10 in Blocking Buffer. After several rinses with phosphate buffer (200 mg/L KCl, 200 mg/L KH_2_PO_4_, 8000 mg/L NaCl, 1150 mg/L Na_2_HPO_4_)- 0.1%Tween), the membrane was incubated for two hours at r.t. with a secondary anti-human IgE antibody conjugated to the enzyme alkaline phosphatase (Mouse Anti-Human IgE Fc-AP, clone B3102E8; Southern Biotech, Birmingham, AL, USA) diluted 1:500 in Blocking Buffer. The membrane was washed again in PBS-T and the immunoreactive signals were detected by a colorimetric reaction in presence of 5-bromo-4-chloroindolyl phosphate/Nitroblue Tetrazolium and 0.1 M Tris, 0.5 mM MgCl2 (pH 9.5) (Alkaline Phosphate Conjugate Substrate Kit, Bio-Rad). Stained membranes were scanned with a ChemiDocTM XRS^+^ Molecular Imager (Bio-Rad). SDS-PAGE protein molecular weight standards, to monitor the run, and proteins from biscuits containing egg and milk ingredients (“BuoniCosì”, Galbusera), as positive control, were loaded. Analyses were performed on two different portions of Magretti biscuits previously administered to enrolled patients. (Table S2). Unspecific signals due to the secondary antibody were identified performing immunoblots without patients’ sera or with pooled sera of non-allergic pediatric patients. A Relative Volume Quantity analysis was performed by a densitometric analysis of blots using Image Lab Software (version 6, Bio-rad). Lane of positive control (biscuits containing egg and milk) was set as reference, and for all other lanes of the blot numerical values relative to the reference were calculated as ratio of the background-adjusted lane volume divided by the background-adjusted reference volume.

#### 2.6.2. LC-ESI-MS/MS Analysis

Two different procedures were followed, route 2.A or 2.B (Figure 1), in order to apply different workflows and instrumental platforms with the aim to validate the final results.

Method 2.A: One g of biscuits’ powder was combined with 5 mL of Hexane, left shaking at high speed for 15 min at r.t., centrifuged at 4000 g for 20 min, and the supernatant discarded. This lipid removal procedure was repeated once again and samples were dried by evaporation using a flow of nitrogen. Four mL of extraction buffer [50 mM Trizma base, 2 M Urea, 1% (*w/v*) Octyl ß-D-glucopyranoside] were added to each defatted sample, which was shaken at high speed for 1 h at r.t. After centrifugation, 500 μL of supernatant (protein extracts) were reduced for 60 min, shaking at 1000 rpm at 60 °C with 5 mM (final concentration) Tris-(2-carboxyethyl)-phospine, incubated with 25 μL of 100 mM cysteine blocking reagent (Methyl methane-thiosulfonate) for 15 min at r.t., diluted with 425 μL of digestion buffer (100 mM Ammonium bicarbonate, 5 mM Calcium chloride), and digested with 20 μg Trypsin TPCK treated (SCIEX, Redwood City, CA, USA) at 37 °C for 16 h. The reaction was stopped with 30 μL of Formic acid (FA) and the digested samples were filtrated using a centrifugal filter unit with 10 kDa MWCO (Merck Millipore). The filtrates were further analysed by mass spectrometry. In order to obtain a calibration curve spanning the concentration range 5–50 part per million (ppm, defined as μg of allergenic ingredient per g of matrix) for quantitative analysis, biscuits fortified with allergen commodity (egg powder and skim milk powder, certified reference material BCR-685, Sigma-Aldrich, Milan, Italy) were prepared and processed as samples.

Digested samples were analyzed using a micro-LC-ESI-Triple Quadrupole (TQ) platform: a M3 MicroLC-TE System interfaced with a QTrap6500^+^ mass spectrometer equipped with an IonDrive Turbo V Ion Source (TurboIonSpray probe with 50 μm i.d. electrode; Sciex). In this case, 10 μL of tryptic peptides (~40 μg) were injected onto a ChromXP C18 trap column cartridge (5 μm × 10 mm, 120 Å, 300 μm i.d.; Sciex) for pre-concentration and desalting at a flow rate of 50 μL/min, and subsequently separated using a HALO peptide-ES C18 column (300 μm × 150 mm, 160 Å, 2.7 μm; Advanced Materials Technology, Wilmington, DE, USA) maintained at 40 °C. Mobile phase A was H_2_O + 0.1% FA, and mobile phase B was 0.1% FA in acetonitrile (ACN). Peptides were separated by linear gradient of 2–40% mobile phase B over 11 min at a flow rate of 10 μL/min, followed by a 2 min rinse with 98% mobile phase B. The column was re-equilibrated at the initial conditions for 5.4 min. The QTrap mass spectrometer was operating in positive ESI High Masses Multiple Reaction Monitoring (MRM) mode (Unit Resolution on Q1 and Q2); the data were acquired using Analyst (version 1.6.3, Sciex). Source/Gas parameters were: 20 psi Curtain Gas; Medium Collision Gas; 5500 V IonSpray Voltage; 150 °C Temperature; 35 psi Ion Source Gas 1; 20 psi Ion Source Gas 2, and instrumental settings optimized for each individual milk and egg peptide marker are reported in Table S3. Analyses were performed on two different portions of Magretti biscuits used for the OFC and SPT (Table S2).

Method 2.B: A subset of samples (from cookies administered to child patients namely n. 8, 9, 11, 13–14, 16–21, 23, 25–28) underwent a different sample preparation workflow and a MRM method was built up on a different platform: an LX50 UHPLC pump provided with an autosampler and an ESI interface connected to a QSight 220 TQ mass spectrometer (PerkinElmer Inc., Waltham, MA, USA) as already published [20].

Instrumental settings optimized for each individual milk and egg peptide marker are reported in Table S4. All analyses were performed in triplicates.

Allergen-free and incurred biscuits at the highest level of 300 μg allergenic ingredient/g matrix were produced at laboratory scale according to the procedure described elsewhere [21]. Lower concentration levels covering the calibration range as previously indicated in route 2.A were produced from these two stock samples by appropriate dilution with blank matrix powder. The allergen-free biscuit digest was fortified with increasing amount of synthetic standard peptides (GenScript, Piscataway, NJ, USA) specific for milk and egg allergens (in the range 0.0125–0.2500 μg/mL) and calibration curves were prepared by plotting the signal of each candidate peptide against the inclusion level in the biscuit sample. All extracts were submitted to the workflow previously described before its injection (10 µL) in duplicate in the QSight equipment.

For quantification purposes, each synthetic peptide with peptide concentration (expressed as μg/mL) was first converted in protein molarity, assuming that full digestion of the protein took place and then a proper conversion factor was applied for the calculation taking into consideration the mass/volume ratio used for protein extraction.

Quantitative analysis on data obtained by QTrap mass spectrometer was performed by MultiQuant software (version 3.0.2, Sciex) applying MQ4 algorithm for peak integration (minimum Gaussian smooth width of 1 point) and data processing. Calibration curves were generated by plotting peak areas against allergen commodity concentrations, with 1/x fitting. In particular, calibration points were produced spanning one order of magnitude concentration range expressed as µg allergenic ingredient/g matrix.

Peak integration and data processing on QSight 220 spectrometer MS data was performed by using 3Q Simplicity software (version 1.4, Perkin Elmer) applying Moving Average algorithm for peak integration (minimum Gaussian smoothing of 5 point).

The reporting units were converted into total proteins of allergenic ingredient (µg/g) assuming 35.39% and 48.00% of total protein content for milk and egg, respectively, in accordance with what reported by USDA.

### 2.7. Statistics

As our objective was to verify whether Magretti may be considered technically hypoallergenic for a population of children allergic to baked egg or milk, our sample size was calculated according to the American Academy of Pediatrics (AAP) guidelines for clinical testing of hypoallergenic formulas [22]. The number of subjects needed to project with 95% confidence (one-sided interval) that less than 10% of infants will react to the product is 29 if no clinical reactions are observed, and 43 if one clinical reaction is observed. These sample size estimates were derived based on binomial distribution techniques using Wald’s method for deriving confidence intervals for single proportions.

The analysis on the primary outcome parameter was a per protocol (PP) analysis. Additional outcomes were obtained on Full Analysis Set (FAS) based on the intention-to-treat (ITT) assumption. Quantitative parameters have been summarized by descriptive statistics (arithmetic mean, standard deviation, minimum, median, and maximum) and qualitative parameters by frequencies and percentages. Categorical variables have been presented using non-missing observations and percentages. Denominators for calculation of percentages have been taken as the number of subjects with non-missing observations in the specified population unless otherwise stated. Continuous variables have been presented using number of subjects in the analysis population (N), number of subjects with non-missing observations (n), mean, standard deviation (abbreviated as “SD” in statistical tables), median, minimum (Min) and maximum (max). Unless stated otherwise, statistical tests have been conducted as two-sided at a level of significance *p* = 0.05. *p*-values for difference from baseline have been calculated using paired t-test.

Statistical analyses were performed using the SPSS statistical software, version 19.0 (SPSS Inc., Chicago, IL, USA).

## 3. Results

### 3.1. Clinical Characteristics

Over the four-year period considered, among 379 children confirmed with milk/egg allergy at our hospital, 152 patients were reactive with baked milk/egg. In this case, 41 of them met the severity criteria. Nine patients were excluded: two did not accept the confirmatory challenge, six returned negative at DBPCFC, and one had celiac disease.

The test was proposed to 32 children, positive at entry OFC with Plasmon (23) and/or Pavesini (12). Two families did not agree to participate in the study, and one withdrew her consent on the day of the test when the child refused to perform the blood test. Hence, 29 children (17 male and 12 female, median age 6.97 years, range 0.67–16.75 years) were included in the study population (Table S5). Their clinical characteristics are reported in Table S6. In this case, 25 were allergic to milk (21 had positive SPT to milk-containing biscuits), 19 to egg (11 SPT-positive to egg-containing biscuits); 15 patients were allergic to both foods, three of whom returned positive to both baked egg and milk (Table 2).

Five out of the 29 included patients, were not exposed to confirmatory food challenges due to recent anaphylaxis (three in the group under two years, one in the group 5–13 years and one in the group 13–18 years). The remaining 24 underwent DBPCFCs with baked milk (15 cases), baked egg (five cases), or both (four cases). From these challenges, the mean thresholds of reactivity were 116.3 (± 107.6) and 128.3 (± 96.7) mg protein for milk and egg, respectively.

No patient presented any symptoms at any challenge time during OFCs with the tested product. Equally negative were all skin tests: no patient resulted SPT-positive to Magretti.

### 3.2. Determination of Patient Serum Binding to Egg and Milk Allergens Contained in Biscuits

**Figure 2:**
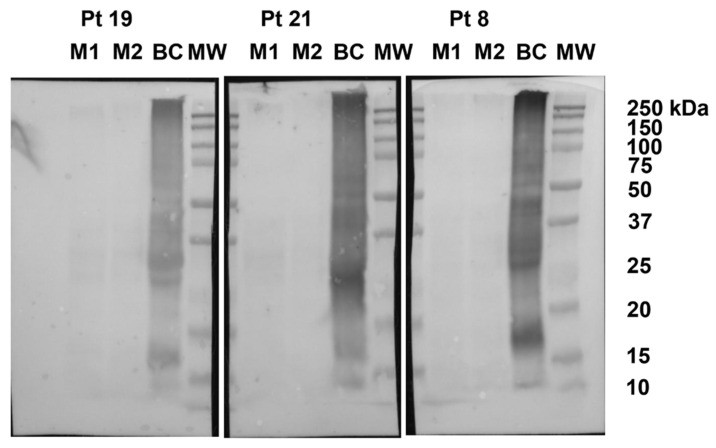
Representative immunoblots. Proteins extracted from two doses (M1 and M2) of biscuit tested on patients (Pt) 19, 21, and 8, blotted with the corresponding patient serum. Proteins extracted from biscuits containing egg and milk ingredients (BC) were loaded as positive control. MW: protein molecular weight standards.

The mean total soluble protein recovery was 7.7 mg per gram. Immunoblots did not show reactions between serum of 18 patients and proteins contained in biscuits (Figure 2). In biscuits from patients 3, 6, 12, 15, 18, 20, 22, 23, 25, 26 and 29, non-specific signals were detected accounting for 30.12% of the positive control.

### 3.3. Determination of Cow’s Milk and Hen’s Egg Allergen Levels in Commercial “Milk and Egg Free” Biscuits by Targeted Mass Spectrometry Methods

Using method 2.A, a mean of ~10 mg of protein was extracted from 1 g of biscuits. About 4 mg of each protein extract was subjected to reduction, alkylation and protein digestion. In method 2.B a different sample preparation workflow was used. In both methods, peptides from milk αS1-casein and egg ovalbumin allergens were used as marker proteins by MRM analysis.

To evaluate the sensitivity of the method, matrix-matched calibration curves, obtained by fortifying biscuits with increasing amounts of allergenic ingredients to cover the range 5–50 mg of allergens/kg of matrix, were built up for each milk and egg allergen marker selected. By interpolating the data of the matrix matched calibration curve, the linearity over the concentration range investigated for all the markers monitored was verified, with a correlation coefficient always better than 0.98. Finally, limit of detection (LOD) and quantification (LOQ) values were, respectively, calculated as 3 and 10 times the standard deviation of the line intercept divided by the slope of the calibration equation for both methods.

Table 3 reports the calculated LODs for methods 2.A and 2.B depending on the instrumental platform used and the selected transitions monitored. Using method 2.A, we were able to detect traces of milk and egg allergens at the lowest range of 0.6 µg _tot prot_/g _matrix_ for milk and egg. Method 2.B was able to quantify milk and egg allergen in biscuits at the lowest level 0.1 and of 0.3 µg _tot prot_/g _matrix_, respectively.

Once the methods had been optimized, they were applied to Magretti to verify their allergen contamination at the lowest level offered by the method. The two methods showed a good agreement of the results obtained. None of the analyzed samples was found contaminated with milk and egg according to the sensitivity offered by the MS method (Table 3).

## 4. Discussion

Allergy to baked milk/egg occurs in a minority of patients allergic to the respective native foods. In previous experiences, this proportion is around 30% [23,24,25,26]. On the total allergic patients enrolled in this study 40.1% of children with milk/egg allergy were reactive to baked foods. This higher percentage may reflect a high severity of our caseload, afferent to a third-level hospital with a catchment area corresponding to the entire Italian nation. To focus on the most severe forms of baked egg/milk allergy, we applied an arbitrary definition of severity including clinical data and sensitization parameters. Basing on this, one fourth was defined severe. Most of the severe milk/egg allergic patients (21/29) had a history of anaphylaxis. In these highly selected patients, we found thresholds of 116.3 and 128.3 mg protein for milk and egg, respectively, at DBPCFC. As in previous studies these thresholds ranged between 0.6 and 150 mg for milk and between 0.65 and 200 mg for egg [27], our patients severely allergic to baked foods do not present thresholds below those allergic to milk/egg, confirming previous data [13,28,29].

In the studied population, no signs or symptoms of allergic reactions were recorded at OFC with the milk/egg free biscuits. In the light of quantitative assessments, appears that Magretti does not pose a real danger from possible accidental contamination.

In this model, can we predict the absence of risk simply using an accurate quantification of milk/egg allergens contained in foods? Probably we can.

According its labelling, Magretti may contain up to 5 mg/kg milk/egg protein, an amount exceeding the VITAL threshold by 20%. In principle, they are likely to induce allergy in up to 1% of milk/egg allergic individuals, and more in patients with severe food allergy. By analysing biscuits by LC-ESI-MS/MS methods, no sample was found contaminated at levels close to the 5 mg/kg indicated by the producer, corresponding, respectively, to 1.77 µg milk or 2.40 µg egg proteins/g of matrix. In addition, the VITAL 3.0 reference dose for milk and egg was not even remotely approached. We found a milk and egg protein content < 0.6 µg /g matrix of milk (method A), <0.1 µg /g matrix of milk and <0.3 µg/g matrix of egg protein (method B). As we administered between 31.75 and 45.75 g Magretti biscuits during OFCs, our patients were exposed to a maximum of 3.18/4.58 µg milk and a maximum of 9.53/13.64 µg egg protein, respectively. These values are much lower than the VITAL 3.0 limit of 200 micrograms for the same amount of milk or egg, under which precautionary cross-contact statement is not required. We can therefore confidently assume that mass spectrometry is able to confirm the absence of protein allergens up to a calculated level thus assuring a high level of safety for our patients. If this were the case, biscuit producers could be advised not to adopt any PAL for products containing such tiny amounts of milk or egg proteins: the risk would be far lower than the 1% predicted by VITAL grids.

Among omics sciences, proteomics and particularly MS-based proteomics are gaining a steadily increasing interest by the scientific community thanks to the recent and rapid technological advances made: up-to-date mass spectrometers have risen unprecedented specificity, sensitivity and capability to perform multiplexing analysis for allergen determination through their peptide/protein markers. For food control, MS-based proteomics approaches are currently applied for the detection and quantification of allergenic ingredients intended ads contaminants. In this regard, mass spectrometrists are making great efforts to develop allergens accurate quantification methods; MS strength lies in its ability to unequivocal identify allergens and multiplex the analyses, opening to the quantification of several allergenic proteins in complex matrices within a single LC-ESI-MS/MS run with high analytical confidence [30,31].

Aware of the technical difficulty of the proteomic methods, and of the likely bias among the different laboratories, we designed this study including analysis carried out in two analytical laboratories using different analytical platforms in order to compare the results originated by different analytical strategies and monitoring specific peptides/transitions for each selected allergen. On this regard, the two applied proteomic methods (2.A and 2.B, Figure 1), based on Triple Quadrupole mass spectrometry detection, followed a different analytical workflow and used different peptide transitions as quantifier ions, but did not provide significantly different values (Table 3). We infer that the standardization of proteomic methods may allow the analytic window necessary for an almost complete exclusion of allergic risk. By contrast, immunoblotting is in our hands too coarse to be able to contribute to the necessary information in this field, because it is burdened with interference errors.

Ideally, this study should have been conducted on ‘very small amounts of egg/milk reactors’. As our first challenge dose was of 3.5 mg of milk protein or 5.2 mg of egg protein, we are not including patients positively allergic to very small amounts. Reaction to traces of milk and egg is an exceptional phenomenon, happening in less than 1% of food-allergic patients by VITAL definition. In order to be able to transfer the same study to a population of trace-allergic patients, it would be necessary to have a basic series of 2900 patients allergic to baked milk or egg, which is not available to us and largely exceeds any caseload ever published.

A second limitation is that we were not able to stratify patients based on a shared definition of food allergy severity. In the present situation, a precise classification of the different phenotypes of food allergy in a homogeneous way between different caseloads is not possible. The imminent definition of severe food allergy could help fill this unmet need [32,33,34].

A third limitation of our study could be the use of open OFCs in the evaluation of reactivity to the tested biscuit. The results deriving from this type of OFC can be different those of DBPCFC in diagnostic terms. However, it has been shown that they can be overlapping in terms of evaluation of food allergen dose distribution [35].

A further limitation of this study is that the MS-based approaches we used for the detection and quantification of allergenic ingredients are able to detect an amino acid sequence of the allergenic protein, but not necessarily the epitope recognized by immune system. Thus, it may be theoretically possible that they miss small parts of allergenic proteins. On the other hand, the presence of a peptide marker will definitely imply the presence of a milk or egg allergen traces giving rise to assume that a cross-contact with the allergen sources has occurred. According to its well-known selectivity and sensitivity, MS have the power to overcome immunoassays (enzyme-linked immunosorbent assay, ELISA) and PCR-based techniques, the historically most adopted methods for allergens detection and quantitation. Specifically, although ELISA methods boost a general high sensitivity, they still encounter disadvantages such as cross-reactions with food matrix, limited reproducibility, variable specificity of antibodies in the commercial kits, lack of standard reference materials for some allergens and missing multiplexing detection ability [36].

## 5. Conclusions

Due to the technological limitations, the current approach to PAL relies on a non-analytic-based risk assessment. As in our study the sensitivity of MS proteomic largely exceeding the limits recommended by the VITAL grid, we conclude that an accurate quantification of tiny amounts of protein in complex foods, in combination with population clinical studies, deserves the potential to establish exact reference doses below which no reactions could be exerted even in the most sensitive individuals.

When proteomic determinations show that the controls carried out at the level of the production and distribution chain are sufficient to avoid this risk for the tested product, the clinician may be authorized to exempt children allergic to milk and egg from observing PAL.

## Figures and Tables

**Table 1:** Magretti biscuit composition.

Ingredient	Quantity
Type 2 soft wheat flour	63%
Sugar	
Cereal flour	10% (corn 5%, barley 5%, on the finished product)
High oleic sunflower oil	10%
Honey	
Barley malt and corn extract	
Raising agents	ammonium acid carbonate, disodium diphosphate, sodium hydrogen carbonate
Whole sea salt	0.5%
Emulsifier	
Aromas	
Allergy warning	The recipe does not contain milk and eggs. It cannot be excluded that any traces of these allergens are present, in any case less than 5 mg/kg. The product can also contain soy, hazelnuts and other nuts; therefore, it is not suitable for consumption by people allergic to these substances.

**Table 2:** Patients’ sensitization to milk and egg (number of patients, #; mean ± SD of sensitization values).

	sIgE > 0.35 kUI/L		c-SPT > 3 mm		ffSPT > 3 mm	
	#		#		#	
Milk	23	48.4 ± 39.7	24	10.3 ± 5.3	25	11.4 ± 4.5
Casein	19	49.1 ± 45.4	15	9.7 ± 4.9		
Egg white	15	27.1 ± 46.2	19	7.6 ± 2.5	15	7.0 ± 2.2
Egg yolk	9	32.4 ± 45.2	14	9.0 ± 3.5	10	8.0 ± 1.2
Baked milk biscuit (Plasmon)					21	7.2 ± 3.8
Baked egg biscuit (Pavesini)					11	4.7 ± 1.2
Magretti-Frollini con orzo e mais biscuit					0	

SD: standard deviation; sIgE: specific IgE; c-SPT: commercial skin prick test; ffSPT: fresh-food skin prick test; n.d.: not determined.

**Table 3:** Results of MRM experiments referred to matrix-matched calibration curves produced in fortified biscuits (route 2.A and route 2B) employing synthetic peptides for quantification (in route 2.B).

Allergen	Protein	Quantifier Peptide (*m/z*)	Product ion (*m/z*)	LOD µg _tot protein_/g _matrix_	R^2^	Route
Milk	α-S1-Casein Bos d9	634.4 (YLG)	991.6	0.63	0.99	2.A (QTrap 6500^+^)
		692.9 (FFV)	991.4	0.10	0.99	2.B (QSight 220)
Egg	Ovalbumin Gal d2	844.4 (GGL)	666.3	0.61	0.98	2.A (QTrap 6500^+^)
		592.1 (ISQ)	858.9	0.30	0.99	2.B (QSight 220)

*m/z*: mass-to-charge ratio of the peptide ion and product; LOD: limit of detection calculated as 3 × SD of the intercept calculated on the matrix matched calibration curve and whose goodness of the linear interpolation is reported by R2.

## Data Availability

Dataset is available on request.

## Supplementary Materials

The following are available online at https://www.mdpi.com/article/10.3390/nu13124540/s1, Table S1: Doses of biscuit administered to children in the experimental challenge, Table S2: Correspondence amongst Magretti doses tested on patients by OFC and SPT and samples subjected to proteomic experiments, Table S3: QTrap 6500+ MS compound parameters of milk α-S1-Casein and egg albumin detected ions, Table S4: QSight 220 MS compound parameters of milk α-S1-Casein and egg albumin detected ions, Table S5: Demographic data of the patients, Table S6: Clinical characteristics of the patients.
