# Supplementary material for: Threshold of Reactivity and Tolerance to Precautionary Allergen-Labelled Biscuits of Baked Milk- and Egg-Allergic Children
Source: Nutrients. 2021 Dec 18;13(12):4540. doi: 10.3390/nu13124540 (PMC8709061; doi:10.3390/nu13124540)
Supplement: Supplementary file 1 [file nutrients-13-04540-s001.zip › nutrients-1493168-supplementary.pdf]

nutrients MDPI

Table S1. Doses of biscuit administered to children in the experimental challenge. All patients were challenged with six growing doses. The 7th and 8th dose were higher for older patients, reflecting the different reasonable portion sizes. Plasmon Primimesi contain 1.3 g milk protein per 100 g product. Pavesini contain 2.088 g egg protein per 100 g product.

All patients	Dose #	Dose (g)	Total dose (g)	Milk (mg proteins)	Total milk (mg proteins)	Egg (mg proteins)	Total egg (mg proteins)
	I	0.25		3.25		5.2	
	II	0.5		6.5		10.4	
	III	1		13		20.9	
	IV	2		26		41.8	
	V	4		52		83.5	
	VI	8	15.75	104	204.75	167	328.8
**Age group**							
< 5	VII	16	31.75	208	412.75	334.1	662.9
6 to 9	VII	20	35.75	260	464.75	417.6	746.5
10 to 13	VII	25	40.75	325	529.75	522	850.9
14 to 18	VII	10	45.75	390	594.75	208.8	955.3
	VIII	20				417.6	

Table S2. Correspondence amongst Magretti doses tested on patients by OFC and SPT and samples subjected to proteomic experiments. A portion of two of the different doses administered to each patient was evaluated. Fifty-eight biscuit samples were analyzed.

**Magretti biscuit**			**Patient #**																												
			1	2	3	4	5	6	7	8	9	10	11	12	13	14	15	16	17	18	19	20	21	22	23	24	25	26	27	28	29	**Total**
**Clinical tolerance test**	**SPT**			X		X									X					X					X		X				X	7
	**OFC dose**	**I**			X					X			X					X					X					X				6
		**II**						X				X				X						X			X	X	X					7
		**III**				X								X				X	X		X		X						X	X		8
		**IV**	X						X			X					X			X				X								6
		**V**	X					X		X				X		X						X							X			7
		**VI**		X			X		X						X											X		X			X	7
		**VII**			X		X				X		X				X				X			X						X		8
		**VIII**									X								X													2
		**Sum**	2	2	2	2	2	2	2	2	2	2	2	2	2	2	2	2	2	2	2	2	2	2	2	2	2	2	2	2	2	**58**

Table S3. QTrap 6500⁺ MS compound parameters of milk α-S1-Casein and egg albumin detected ions.

Allergen	Protein	Peptide sequence	Precursor ion m/z (charge)	Product ion *m/z*	Product ion Series	Fragment ion identification	DP	CE	EP	CXP	Time
Milk	α-S1-Casein Bos d9	YLGYLEQLLR (YLG)	634.4 (+2)	991.6	y_8^{+1}	Quantifier	75.5	33.4	10	14	50
				249.16	a_2^{+1}	confirmative	75.5	29.4	10	14	50
Egg	Ovalbumin Gal d2	GGLEPINFQTAADQAR (GGL)	844.4 (+2)	666.34	y_{12}^{+2}	Quantifier	88.5	38.4	10	14	50
				1121.53	y_{10}^{+1}	confirmative	88.5	40.4	10	14	50

m/z: mass-to-charge ratio; DP: declustering potential (V); CE: collision energy (V); EP: entrance Potential (V); CXP: collision cell exit potential (V); Time: dwell time (ms)

Table S4. QSight 220 MS compound parameters of milk α-S1-Casein and egg albumin detected ions.

Allergen	Protein	Peptide sequence	Precursor ion *m/z* and (charge)	Product ion		Fragment ion identification	EV	CCL2	CE
				m/z	Series				
Milk	α-S1-Casein Bos d9	FFVAPFPEVFGK (FFV)	692.9 (+2)	991.4	y_9^{+1}	quantifier	17	-168	-26
				1090.4	y_{10}^{+1}	confirmative	25	-176	-26
				920.3	y_8^{+1}	confirmative	15	-168	-26
Egg	Ovalbumin Gal d2	ISQAVHAAHAEINEAGR (ISQ)	592.1 (+3)	858.9	y_8^{+1}	quantifier	30	-204	-29
				778.5	y_7^{+1}	confirmative	42	-128	-31
				545.9	y_5^{+1}	confirmative	20	-200	-40

m/z: mass-to-charge ratio; EV: entrance voltage (V); CCL2: collision cell lens 2 (V); CE: collision cell energy (V)

Table S5. Demographic data of the patients.

	#	Min	Max	Mean (SD)
**Age (months)**		0.67	16.75	6.97 (4.77)
< 2 years	4			
2-5 years	10			
6-12 years	11			
12-17 years	4			
**Male / Female**	17/12			
**Race**	Caucasian	28	Asian	1
**Anthropometrics**				
Weight (kg)		7.700	61.500	26.33 (11.53)
Height (cm)		67	171	117.60 (30.71)
BMI		12.26	22.60	16.99 (2.47)

BMI: Body Mass Index; SD: standard deviation

Table S6. Clinical characteristics of the patients.

**Symptom**	#
Anaphylaxis	21
Atopic Dermatitis	15
Asthma	12
Allergic rhinitis	16
**Sensitization to other food allergens**	**23**
Tree nuts	5
Peanut	4
Wheat	4
Codfish	6
Kiwifruit	6
**Sensitization to inhalants**	**26**
Pollens	14
Dust mites	16
Cat, dog dander	12

Table S7. Patients' sensitization to milk and egg (mean ± SD).

	sIgE (kUI/L)	c-SPT, diameter (mm)	ffSPT diameter in (mm)
Milk	48.4 ± 39.7	10.3 ± 5.3	11.4 ± 4.5
Casein	49.1 ± 45.4	9.7 ± 4.9	
Egg white	27.1 ± 46.2	7.6 ± 2.5	7.0 ± 2.2
Egg yolk	32.4 ± 45.2	9.0 ± 3.5	8.0 ± 1.2
Baked milk biscuit (Plasmon)			7.2 ± 3.8
Baked egg biscuit (Pavesini)			4.7 ± 1.2

sIgE: specific IgE; c-SPT: commercial skin prick test; ffSPT: fresh-food skin prick test; SD: standard deviation
